# The development and validation of a clinical measurement tool for fear of recurrence and progression in cardiac patients

**DOI:** 10.1038/s41598-026-40353-5

**Published:** 2026-03-16

**Authors:** Sarah T. Clarke, Michael R. Le Grande, Barbara M. Murphy, Robert Hester, Alun C. Jackson

**Affiliations:** 1 Australian Centre for Heart Health, Melbourne, Australia; 2 Melbourne School of Psychological Sciences, University of Melbourne, Parkville, Australia; 3 Centre on Behavioral Health, University of Hong Kong, Pokfulam, Hong Kong Republic of China

**Keywords:** Fear of recurrence and progression, Cardiac rehabilitation, Cardiac recovery, Psycho-cardiology, Psychological measurement, Cardiology, Diseases, Health care, Medical research

## Abstract

One in two cardiac patients fear having another heart event or their cardiac condition worsening. Assessment of fear of recurrence and progression (FoRP) is critical to effective clinical care and chronic disease management. The current study aimed to develop and validate a measure of cardiac-FoRP - The Fear of Cardiac Recurrence and Progression Inventory (FCRPI). Items were generated by reviewing relevant literature and were refined in collaboration with key informants. The item pool (44 items) was administered to 241 adults (mean age *=* 64.52; 50% female) with a history of an acute coronary event, cardiac surgery, or chronic cardiac condition. Exploratory factor analysis identified the underlying factor structure and Rasch analysis assessed fit and reduced the item set. The final FCRPI comprised 29 items across five fear subscales relating to: deteriorating health, requiring further treatment, disengagement and loss of agency, impacts on intimacy, and impacts on work and finance, and two fear response subscales: avoidance and hyperawareness. The FCRPI demonstrated excellent internal consistency, and criterion and discriminant validity, and a clinically significant cut-off score of 39 was identified. The FCRPI offers both research and clinical utility by providing a validated tool to assess cardiac-specific FoRP and guide tailored interventions and patient-centred care.

**Supplementary Information:**

The online version contains supplementary material available at 10.1038/s41598-026-40353-5.

## Introduction

Fear of recurrence and progression (FoRP) is well-recognised as a primary source of distress among people living with chronic illness. Referring to the fear that a condition may return or worsen along with the consequences this may entail, FoRP is considered a rational and appropriate response to the real threat of disease^1,2^. As such, FoRP is distinct from general anxiety which is typically disproportionate to the actual danger posed^3^. FoRP can represent a clinically significant issue for patients, particularly when it leads to persistent preoccupation and worry, maladaptive coping, functional impairment, and heightened distress^4,5^. In psycho-oncology, clinically significant FoRP has been established as both common and highly burdensome for patients, undermining psychological wellbeing, quality of life, and daily functioning^5–8^. Research in psycho-oncology has seen the development of several FoRP instruments^6^and specific interventions to address and manage FoRP in survivors^9^.

FoRP has more recently gained attention in psycho-cardiology, being increasingly recognised as a key contributor to distress for people living with heart disease^10–14^. Our previous work estimates that one in two cardiac patients experience FoRP, with many describing this experience as highly distressing^11^. Moreover, FoRP appears to persist long after the time of first cardiac event and/or diagnosis^10,11^. Emerging research has demonstrated that cardiac-FoRP is associated with poorer psychological wellbeing, including elevated anxiety, depression, post-traumatic stress symptoms, and sleep disturbances, as well as reduced quality of life, self-care confidence, and wellbeing^10,15–24^.

Despite its clinical relevance, there are currently no cardiac-specific tools to assess FoRP, nor have interventions been developed to manage these concerns in clinical care. Cardiac research to date has largely relied on transdiagnostic measures of FoRP that were designed for use within a range of illness presentations but which have not been validated in cardiac populations^1,25^. One transdiagnostic measure of FoRP has been validated in a cardiac disease population^26^, but has yet to be adopted in cardiac patient research. While transdiagnostic measures are useful for conducting research across a range of disease cohorts, their utility in cardiac research is impeded by the inclusion of items irrelevant to, and the omission of items uniquely relevant to, the cardiac disease experience. For example, cardiac patients commonly report fears related to physical exertion, sudden death, and access to emergency care, as well as specific responses to these fears such as heightened monitoring of cardiac sensations and avoidance of physical activity^11^. These experiences are not comprehensively captured in existing transdiagnostic FoRP measures. The development of a valid and reliable cardiac-specific measure of FoRP, co-designed with this patient group, is critical to facilitating comprehensive research and enabling clinicians to better identify and address FoRP in cardiac patients.

The aim of the present study was to develop and validate the Fear of Cardiac Recurrence and Progression Inventory (FCRPI). The FCRPI will assist health professionals in assessing and characterising fears relating to disease recurrence, progression, and related consequences in cardiac patients, thereby facilitating development of and referral to targeted psychotherapeutic interventions to support cardiac patient recovery.

## Method

The methodology used to develop the FCRPI followed best practice for instrument design^27^as described in detail in the published study protocol^11^. In brief, this process involved five steps:

*Step1*: Literature review to characterise and define FoRP within the cardiac patient population and ensure that no measure already existed to assess this construct.

*Step 2*: Initial generation of items through review of existing measures to identify items relevant to cardiac-FoRP, including (a) FoRP measures designed for use in other illness populations, including transdiagnostic measures and (b) measures of associated cardiac disease-related mental health outcomes such as anxiety, fear, and distress.

*Step 3*: Generation of additional items and validation of existing items through scoping review of literature relating to FoRP experiences in cardiac patients^11^.

*Step 4*: Content validation through review by multidisciplinary investigator group to reach consensus on relevance of items, phrasing of scale instructions, and formatting.

*Step 5*: Generation of additional items and content validation through co-design and collaboration with three key informant groups, with iterative changes made between each consultation. The three groups were: Group 1 - cardiac health professionals (*n* = 17) including cardiac rehabilitation coordinators, nurses, exercise physiologists, psychologists, and psychiatrists; Group 2 - academic experts in psycho-cardiology and health instrument development (*n* = 5); Group 3 - people with lived experience of cardiac disease (*n* = 7).

These five steps resulted in a pool of 44 items. Items were separated into two sections with different response sets. This comprised 31 items addressing a fear or concern and 13 items addressing a response to fear. The full item pool is presented as Supplementary material (Table S1). All items used a four-point Likert-scale. Response options and inventory instructions are presented with the final FCRPI as Supplementary material (S2).

### Participants

Eligible patients were adults, over the age of 18, who had experienced an acute coronary event, undergone cardiac surgery, or had a chronic cardiac condition. Patients who did not have adequate English proficiency to understand the Participant Information and Consent Form (PICF) were excluded. Participants self-selected to participate. The study was promoted through flyers in healthcare settings, social media posts, and online communications. Study promotions contained information about what participation involved, eligibility, how to register, and contact information.

### Measures

In addition to the FCRPI item pool, several measures were administered for descriptive and validation purposes. A full description of additional measures is provided as Supplementary material (S1). In brief, demographic, psychosocial, and clinical information was collected, including a question regarding COVID-19 concern which was used to examine discriminant validity. Other validation measures included a Visual Analogue Scale (VAS) for FoRP, the Fear of Progression Questionnaire Short Form (FoP-Q-SF)^25^, the Cardiac Distress Inventory Short Form (CDI-SF)^28^, Mishel’s Uncertainty in Illness Scale - Community Form (MUIS-C)^29^, the Patient Health Questionnaire-9 (PHQ-9)^30^, the Generalised Anxiety Disorder Instrument (GAD-7)^31^, and the PTSD Checklist for DSM-5 (PCL-5)^32^.

### Procedure

Participants were recruited between May 2023 and July 2024. Participants completed the PICF and questionnaire pack online available via the Research Electronic Data Capture (REDCap) platform^33^. Access to the questionnaire was obtained by scanning a QR code on physical flyers or by following the link from online posts. Where participants were unable to access a device or the internet, a hard-copy questionnaire pack was mailed (*n* = 5) and returned by post. No identifying information (name, address, date of birth) was collected. The questionnaire took approximately 20 minutes to complete. The study was endorsed by the Australian Cardiovascular Health and Rehabilitation Association (ACRA) and Heart Support Australia and was promoted through their communication avenues. The study was approved by The University of Melbourne Human Research Ethics Committee (reference number: 2023–25413-39497-5) and conducted according to the guidelines of the Declaration of Helsinki.

### Data analysis

Prior to analysis, data were cleaned to remove duplicates identified based on identical demographic information. In the case of duplicates, the first or most complete entry was retained unless the participant reported further cardiac events, surgeries, or diagnoses, in which case the most recent entry was retained. The minimum data set for inclusion in analysis was completion of the FCRPI item pool. Statistical analysis was modelled on the methodology utilised in the design and validation of the Cardiac Distress Inventory (CDI)^34^. This included four stages.

### Stage 1. Exploratory factor analysis (EFA)

EFA was used to establish the factors of the FCRPI. EFA was conducted using Jamovi (v2.6.44) and SPSS (v.27). Prior to EFA, multicollinearity was assessed through investigation of bivariate correlations, where a correlation > 0.8 indicated potential multicollinearity, in which case one of the two items was removed^35^. Items with low communalities (< 0.4) were also removed^36^. The suitability of data for EFA was assessed by Kaiser-Meyer-Olkin (KMO) measure of sampling adequacy (MSA) > 0.8, Bartlett test of sphericity p-value of χ2 test < 0.05, and inspection of the scree plot^35^. The optimal number of factors was informed from parallel analysis of remaining items^37^. Due to the ordinal levels of items, EFA results were evaluated using both the polychoric and correlation matrices^38^. As minimal differences were identified between the two solutions, the correlation matrix was used for ease of interpretability. Minimum residual was used to estimate the factor structure since it gives robust estimates even for items with skewed distribution, which is a common feature of questionnaire items^39^. Several non-orthogonal rotation techniques were tested to assess best model fit in consideration of clinical and theoretical interpretation of factor loadings, with oblimin rotation ultimately being chosen^35^. Variable loadings < 0.32 were supressed to assist interpretability of the EFA solution. Items that did not load on any factor above 0.32 were removed, and the factor loadings reassessed^40^. Model fit was further assessed using multiple tests whereby good model fit was indicated by standardized root mean square residual (SRMS) < 0.08, root mean square error of approximation (RMSEA) < 0.06, and Benteler’s comparative fit index (CFI) and Tucker-Lewis Index (TLI) > 0.90^41^.

### Stage 2. Rasch analysis

Rasch analysis was used to reduce the number of items in the FCRPI by assessing item fit in an iterative process within each factor identified from the EFA. Rasch analysis was conducted using WINSTEPS software (v.5.5.1). Since item responses were all the same format (0,1,2,3), all analyses were conducted using the Rating Scale Model (RSM) in preference to the partial credit model. At each iteration item deletion occurred until model fit improved in criteria with Rasch assumptions and parameters. Rasch analysis assumes unidimensionality, which was assessed using point measure correlations, principal components analysis of the Rasch residuals (PCAR), and Wright’s Unidimensionality Index. Point measure correlations in the positive direction indicated that the real observations agree with the predicted Rasch unidimensional model^42^. PCAR examined whether the residuals of the unidimensional model represented random noise or a potential second dimension. Unidimensionality was assumed where the PCAR suggested that ≥ 40% of variance was explained by the observed data, and the eigenvalue in the first residual contrast was < 2. Where the eigenvalue of the first residual contrast was > 2, PCAR loadings were examined to identify the meaningfulness of the potential second dimension^43^. Wright’s unidimensionality index was calculated by dividing the person separation index using real standard errors by the person separation index using model standard errors whereby a value ≥ 0.9 was indicative of unidimensionality and ≤ 0.5 suggested multidimensionality^44^. Rasch analysis also assumes local independence^45^, which was assessed through inspection of residual correlations, whereby residual correlations > 0.3 may represent local dependence or the existence of a second dimension^46,47^. Monotonicity was evaluated by ensuring that that there were at least 10 observations for each category of the rating scale, that ratings were associated with mean person ability for each scale, and that average category measures advance monotonically up the rating scale^47^.

Item fit was assessed using infit (information-weighted) and outfit (outlier-sensitive) mean square (MNSQ) statistics and standardised z-scores (ZSTD). Items were considered to demonstrate adequate fit with a MSNQ between 0.5 and 1.5^47^. If the MNSQ fell outside of this range, the ZSTD was assessed, whereby ZSTD ≥ 2.0 indicated significant misfit^47^. Item and person separation indices assessed the spread of items and individuals along a continuum of endorsement for each factor, where values ≥ 2.0 represented acceptable separation^48^. Item and person reliability indices were used to assess the reliability of rater observations and consistency of items within a factor, where values ≥ 0.5 was regarded as adequate, ≥ 0.80 as good, and ≥ 0.90 as high reliability^49^.

Differential item functioning (DIF) analysed the extent that items functioned differently across sub-groups of age, sex, and education. A logit difference of at least 0.5 and *p* < 0.05 (Rasch-Welch test) was used to detect problematic DIF^50^.

### Stage 3. Evaluating the psychometric properties of the FCRPI

Evaluation of the psychometric properties of the FCRPI was informed by the Consensus-based Standards for the Selection of Health Measurement Instruments (COSMIN)^51^. Internal consistency of the FCRPI was determined using McDonald’s omega (ω)^52^ and evaluation of the reliability indices from the Rasch analysis. Concurrent validity was established through associations between FCRPI scores, FoP-Q-SF and VAS, as well as related mental health measures (GAD-7, PHQ-9, PLC-5, & MUIS-C). Discriminant validity was established through the association between FCRPI scores and concern about the effect of COVID-19 on heart health.

### Stage 4. Determining the FCRPI cut-off score for clinically significant Cardiac-FoRP

Receiver operating characteristic (ROC) analysis was used to identify a cut-off score for the FCRPI that related to clinically significant distress. While many patients endorse some level of cardiac-FoRP, this cut-off can help to identify more severe distress, which has been proposed as an essential characteristic of clinically significant FoRP^5^. The area under the curve (AUC) estimated the overall discriminative accuracy of FCRPI scores relative to the established CDI-SF cut-off score of ≥ 13 to signify clinically significant distress. AUC ≥ 0.70 was interpreted as acceptable, ≥ 0.80 as good, and ≥ 0.90 as excellent discrimination^53^. The optimal cut-off score was indicated by the highest Youden Index value, representing where the ROC curve maximised both sensitivity and specificity^54^.

### Sample size requirement

Recommendations of sample size for EFA in instrument development is at least five cases for each item being tested^55^. Rasch modelling for exploratory purposes should be based on at least *N* = 100 and preferably *N* = 250^56^.

## Results

### Participants

A total of 321 survey responses was collected, of which *n* = 29 were identified as duplicate cases and removed. Of the 292 participants, all of whom provided informed consent, *n* = 241 completed the minimum data set and were included in the analysis. Participants found out about the study through their health service (*n* = 142), online/social media platforms (*n* = 62), alternative sources such as word of mouth, newsletters, or previous research participation (*n* = 35). Five participants completed the questionnaire using the hardcopy data pack, while the remaining 236 completed the survey online.

Participant characteristics are shown in Table 1. Ages ranged from 26 to 89 (*M =* 64.52, *SD* = 11.23). Time from first event, procedure, or diagnosis to study participation ranged from 1 week to 47 years (*M =* 5.8 years, *SD* = 9.0 years; data provided by *n* = 229). Time from most recent event, procedure, or diagnosis to study participation ranged from 1 week to 25 years (*M =* 1.8 years, *SD* = 3.1 years; data provided by *n* = 225). Our previous study found no significant differences in level of cardiac-FoRP based on time since most recent or first event, procedure, or diagnosis^10^. Endorsement rates for the FCRPI item pool are presented as Supplementary material (Table S1).

**Table 1:** Clinical characteristics (*N* = 241).

	*n* (%)
*Gender*	
Female	120 (50%)
Male	121(50%)
*Age groups (years)*	
< 55	42 (17%)
55–64	70 (29%)
65–74	83 (34%)
≥ 75	46 (19%)
*Country of birth*	
Australia	174 (72%)
United Kingdom	26 (11%)
New Zealand	12 (5%)
Other	29 (12%)
*Country of residence*	
Australia	234 (97%)
Other	7 (3%)
*Education*	
≤ Secondary	44 (18%)
Mid-level (trade)	59 (24%)
University	138 (57%)
*Partnered*	172 (72%)
*In paid employment*	96 (40%)
*Private health insurance*	178 (74%)
*Level of financial strain*	
None	91 (38%)
Slight	61 (25%)
Moderate	60 (25%)
Considerable	21 (9%)
Extreme	8 (3%)
*Cardiac condition/diagnosis*	
ACS/Heart attack	94 (39%)
Heart rhythm disturbance	88 (37%)
Stent/PCI	79 (33%)
Bypass surgery	62 (26%)
Valve surgery	46 (19%)
Heart failure	39 (16%)
Congenital heart disease	21(9%)
Cardiac arrest	18 (7%)
Spontaneous coronary artery dissection	8 (3%)
Takotsubo	4 (2%)
Other (conditions that occurred in *n* ≤ 3)	24 (10%)
*More than 1 event/diagnosis*	139 (58%)*
*At least one cardiac risk factor*	158 (66%)
High blood pressure	112 (46%)
High cholesterol	84 (35%)
Obesity	45 (19%)
Diabetes	38 (16%)
Cigarette smoking	7 (3%)*
*Comorbidity*	152 (63%)
*Attend cardiac rehab*	190 (79%)
*Mental health support following cardiac event/condition*	110 (46%)
*History of any mental ill-health*	94 (39%)
Depression	61 (25%)
Anxiety	59 (24%)

* = *n*(239) due to missing data in *n*(2).

^a^ Top 5 comorbidities included musculoskeletal disease (26% of sample), obesity (19%), sleep apnoea (18%), diabetes (16%), and cancer (8%).

ACS=acute coronary syndrome; PCI=percutaneous coronary intervention.

### EFA

Initial item inspection resulted in four of the 44 items being removed. Two item pairs were identified as having bivariate correlations over 0.8: item 29: ‘*becoming socially isolated*’ and item 31: ‘*becoming more withdrawn from friends’* (*r* = 0.84) and item 32: ‘*avoid activities that make your heart beat faster’* and item 33: ‘*avoid physical exertion’* (*r* = 0.83). Following consultation within the multidisciplinary investigator group regarding clinical relevance, items 29 and 32 were retained while items 31 and 33 were removed. Two items which had communalities < 0.4 were also removed: item 35: ‘*avoid thinking about your heart condition*’ (0.35) and item 39: ‘*avoid medical appointments or check-ups’* (0.30). The remaining 40 items demonstrated high factorability. The KMO indicated an overall MSA of 0.94, indicating high suitability of the data for EFA. Bartlett’s Test of Sphericity was statistically significant, χ2 (780) = 6604.99, *p* <.001, indicating significant correlations between variables. Initial EFA identified 4 items that did not load > 0.32 on any factor and had questionable clinical relevance: item 6: ‘*never getting back to the person you used to be’* where responders may consider loss of a past self rather than fear related to the future; item 7: *‘other people being unable to cope if something happens to you’* where responders may consider the emotions of others rather than their own fears; item 11: ‘*aging’* which is likely too general for assessing cardiac-FoRP, and item 18: ‘*being unable to plan for the future’* which may relate to general uncertainty about the future rather than FoRP. These four items were removed.

Parallel analysis of the remaining 36 items resulted in a seven-factor solution (Table 2) which demonstrated good overall fit (*RMSEA* = 0.06, *SRMR* = 0.03, *TLI* = 0.88, *CFI* = 0.93). The rotated seven-factor model explained 60.1% of variance. Correlations between factors ranged from 0.12 to 0.50 (Table S2), confirming non-orthogonality, and were all positive, reflecting unidimensionality and shared variance across the latent construct of FoRP. The factors were: Factor 1 - deteriorating health (six items), Factor 2 - further treatment (six items), Factor 3 - disengagement and loss of agency (eight items), Factor 4 - impacts on intimacy (two items), Factor 5 - impacts on work and finances (four items), Factor 6 - avoidance (five items), and Factor 7 - hyperawareness (five items). Item 8 ‘*your general health and functioning declining’* loaded on both Factor 1 and 3 but was retained in Factor 1 based on greater clinical relevance. Notably, Factor 4 comprised only two items, which is generally not recommended in EFA due to risk of poor factor stability^36^. However, as the two items were highly correlated (*r* = 0.69, *95%CI* = 0.62–0.75) and not strongly correlated with other scale items (item 5 mean *r* = 0.38; item 14 mean *r* = 0.31), and given the important clinical relevance of the factor content, Factor 4 was retained^57^.

**Table 2:** Factor Loadings.

	Factor 1	Factor 2	Factor 3	Factor 4	Factor 5	Factor 6	Factor 7	Uniqueness
2 Having another heart event.	0.86							0.23
1 Your condition getting worse.	**0.80**							0.32
3 Dying.	**0.66**							0.41
10 Physical activity leading to another heart event.	**0.46**							0.41
8 Your general health and functioning declining.	**0.36**		0.39					0.42
4 Developing other medical problems.	**0.33**							0.52
16 Needing more procedures or surgery.		**0.69**						0.31
15 Needing to go back to hospital.		**0.59**						0.33
17 Needing to take more medications.		**0.54**						0.49
12 Receiving permanent scarring from future heart surgeries or removal of veins.		**0.40**						0.65
9 Not having access to the health care you might need.		**0.35**						0.54
19 Being unable to get help on time.		**0.32**						0.49
29 Becoming socially isolated.			**0.86**					0.15
27 Becoming lonely.			**0.68**					0.36
28 Becoming less of a person.			**0.66**					0.28
13 Losing control over your life.			**0.53**					0.40
30 Becoming a burden to your family.			**0.50**					0.45
26 Becoming unable to engage in activities you enjoy.			**0.47**					0.37
23 Becoming unable to cope effectively.			**0.47**					0.37
21 Becoming unable to fulfil your roles at home.			**0**.**46**					0.33
14 Losing capacity for sexual activity.				**0.67**				0.33
5 Your condition impacting your intimate relationships.				**0.58**				0.32
25 Becoming unable to work.					**0.87**			0.23
22 Becoming unable to fulfil your roles at work.					**0.82**			0.36
24 Becoming unable to support yourself financially.					**0.56**			0.39
20 Being unable to afford future medical treatments.					**0.39**			0.45
37 Avoid going far from home.						**0.81**		0.23
38 Avoid travelling far from your cardiac care team.						**0.78**		0.40
36 Avoid being alone.						**0.61**		0.52
33 Avoid physical exertion.						**0.53**		0.49
34 Avoid stressful situations.						**0.36**		0.60
40 Feel overly aware of your heart in your chest.							**0.86**	0.22
41 Feel overly aware of sensations in your body.							**0.82**	0.27
43 Feel worried that you are having another event when you have chest discomfort, or when your heartbeat is fast or irregular.							**0.56**	0.33
42 Monitor your heart rate.							**0.51**	0.69
44 Feel worried that your condition is getting worse when you notice changes in your body, such as feeling more fatigued, short of breath, or retaining more fluid.							**0.39**	0.44

Seven factor solution from parallel analysis of the 36-item pool. **Factor 1 -** deteriorating health, **Factor 2 -** further treatment, **Factor 3** - disengagement and loss of agency, **Factor 4** - impacts on intimacy, **Factor 5 -** impacts on work and finances, **Factor 6 -** avoidance **Factor 7 -** hyperawareness. Bolded factor loadings signify the factor each item was retained in.

### Rasch analysis

In assessing the assumptions of Rasch, potential issues with dimensionality were identified from the PCAR (eigenvalue in first contrast > 2) for Factors 3 and 5. However, in both factors the percentage of variance explained by the observed data in the PCAR was > 40%, there were positive point measure correlations, and Wright’s Unidimensionality Index was > 0.90, indicating excellent unidimensionality within each scale. Examination of the PCAR factor loading identified three items in Factor 3 (item 27: ‘*becoming lonely’*, item 28: ‘*becoming less of a person’* and item 29: ‘*becoming socially isolated’*; eigenvalue = 2.25, 60.6% of variance explained by observed data) and two items in Factor 5 (item 20: ‘*being unable to afford future medical treatments’* and item 24: ‘*becoming unable to support yourself financially’*; eigenvalue = 2.09, 55.1% variance explained by observed data) which affected dimensionality in their respective scales. An issue was also identified with local independence in Factor 3, with a residual correlation of 0.32 between item 27: ‘*becoming lonely’* and item 29: ‘*becoming socially isolated’*. A potential issue with monotonicity was identified in Factor 2, whereby item 12: ‘*Receiving permanent scarring from future heart surgeries or removal of veins*’ had only 11 responses in the ‘extremely’ category and had a relatively lower correlation (0.65) with the overall factor score. Similarly, in Factor 7, item 42: *‘monitor your heart rate*’ had a correlation of 0.67 with the factor score. Regarding item goodness-of-fit statistics, all item infit and outfit statistics fell within the recommended fit criteria, except for item 42: ‘*monitor your heart rate’* (infit: MSNQ = 1.62, ZSTD = 5.75; outfit: MSNQ = 1.64, ZSTD = 5.79). Considering the potential deviations from Rasch assumptions and parameters, alongside assessment of the separation of items of Wright Map, DIF, item and person separation and reliability indices and the clinical and theoretical utility of items, seven items (items 12, 19, 20, 23, 27, 28, & 42) were removed across the six factors (Table S3 for description of items removed and reasons for removal). Rasch RSM analysis was not run for Factor 4 which, had only two items, rendering it unsuitable for Rasch analysis and measurement of a latent construct^58^.

Following item removal, final Rasch iterations demonstrated improved overall fit in accordance with Rasch assumptions and parameters in each factor. Item fit statistics following removal of items are presented in Table 3. One notable deviation from suggested Rasch parameters was observed following removal of item 42 from factor 7, in which the eigenvalues of the first contrast in PCAR became > 2 (item 40:*‘Feel overly aware of your heart in your chest*’ and item 41: ‘*Feel overly aware of sensations in your body’*; eigenvalue = 2.10, 61.4% variance explained by observed data). The updated Wright Unidimensionality Index = 0.92 and point measure correlations were positive. Considering the lack of deviations in all other assessments of unidimensionality, and that no multidimensionality was identified prior to item removal, the change in eigenvalue may be due to the small number of items remaining in this factor^58^and the similar phrasing between the two item pairs, rather than true multidimensionality. Due to the clinical utility and relevance of the final four items, all four were retained.

**Table 3:** FCRPI factor and item fit.

Factor/Item	Logit measure	SE	Infit MNSQ	ZSTD	Outfit MNSQ	ZSTD
**Factor** **1**. **Deteriorating health**						
8 Your general health and functioning declining.	−0.86	0.11	1.05	0.56	1.13	1.25
2 Having another heart event.	−0.35	0.11	0.75	−3.05	0.74	−2.99
4 Developing other medical problems.	−0.08	0.11	1.17	1.83	1.20	2.07
1 Your condition getting worse.	0.08	0.11	0.85	−1.66	0.85	−1.68
3 Dying.	0.42	0.11	1.03	0.34	0.98	−0.18
10 Physical activity leading to another heart event.	0.79	0.11	1.10	1.07	1.14	1.42
Item Separation = 4.85; Item Reliability = 0.95; Person Separation = 2.31; Person Reliability = 0.84; McDonalds omega = 0.88; Wright Unidimensionality Index = 0.91						
**Factor 2.** **Further treatment**						
16 Needing more procedures or surgery.	−0.38	0.10	0.75	−2.90	0.74	−2.97
17 Needing to take more medications.	−0.36	0.10	1.09	1.00	1.09	1.00
15 Needing to go back to hospital.	0.05	0.11	0.72	−3.32	0.70	−3.49
9 Not having access to the health care you might need.	0.69	0.11	1.43	4.01	1.45	3.94
Item Separation = 3.71; Item Reliability = 0.93; Person Separation = 1.68; Person Reliability = 0.74; McDonalds omega = 0.82; Wright Unidimensionality Index = 0.91						
**Factor 3.** **Disengagement and loss of agency**						
26 Becoming unable to engage in activities you enjoy.	− 0.90	0.11	0.81	−2.12	0.84	−1.56
13 Losing control over your life.	− 0.25	0.11	1.09	0.98	1.12	1.20
30 Becoming a burden to your family.	− 0.18	0.11	1.15	1.52	1.11	1.13
21 Becoming unable to fulfil your roles at home.	0.31	0.11	0.84	−1.75	0.82	−1.99
29 Becoming socially isolated.	1.01	0.12	1.11	1.08	1.00	0.01
Item Separation = 5.43; Item Reliability = 0.97; Person Separation = 2.14; Person Reliability = 0.82; McDonalds omega = 0.88; Wright Unidimensionality Index = 0.93						
**Factor 5. Impacts on work and finances**						
24 Becoming unable to support yourself financially.	−0.60	0.11	1.20	1.80	1.21	1.77
25 Becoming unable to work.	0.09	0.12	0.70	−3.03	0.68	−3.14
22 Becoming unable to fulfil your roles at work.	0.51	0.12	1.11	0.96	1.01	0.14
Item Separation = 3.53; Item Reliability = 0.93; Person Separation = 1.31; Person Reliability = 0.63; McDonalds omega = 0.85; Wright Unidimensionality Index = 0.94						
**Factor 6. Avoidance**						
34 Avoid stressful situations.	−1.26	0.10	1.11	1.13	1.18	1.70
33 Avoid physical exertion.	−0.74	0.10	0.99	−0.06	0.95	−0.46
37 Avoid going far from home.	0.03	0.10	0.85	−1.58	0.81	−1.74
38 Avoid travelling far from your cardiac care team.	0.75	0.11	1.06	0.56	0.84	−1.18
36 Avoid being alone.	1.21	0.12	1.15	1.28	0.90	−0.52
Item Separation = 8.09; Item Reliability = 0.98; Person Separation = 1.63; Person Reliability = 0.73; McDonalds omega = 0.83; Wright Unidimensionality Index = 0.93						
**Factor 7. Hyperawareness**						
44 Feel worried that your condition is getting worse when you notice changes in your body, such as feeling more fatigued, short of breath, or retaining more fluid.	−0.22	0.12	1.26	2.52	1.25	2.50
41 Feel overly aware of sensations in your body.	−0.08	0.12	0.82	−1.95	0.82	−1.97
43 Feel worried that you are having another event when you have chest discomfort, or when your heartbeat is fast or irregular.	0.05	0.12	0.87	−1.40	0.86	−1.56
40 Feel overly aware of your heart in your chest.	0.25	0.12	1.02	0.21	0.99	−0.10
Item Separation = 0.93; Item Reliability = 0.46; Person Separation = 2.16; Person Reliability = 0.82; McDonalds omega = 0.88; Wright Unidimensionality Index = 0.92.						

Item fit statistics and reliability statistics for each factor following item reduction from Rasch analysis. Factor 4 is not included in table as it was not analysed with Rasch.

The final 29-item FCRPI and scoring instructions are presented as Supplementary material (S2). The average total score on the FCRPI was 35.85 (*SD* = 18.90).

### Psychometric properties of the FCRPI

Reliability statistics for each Factor are displayed in Table 3, apart from Factor 4 where ω = 0.82. The overall internal reliability was excellent, ω = 0.95. Concurrent validity of the total FCRPI score was established through large correlations with other FoRP measures and moderate correlations with measures of related mental health outcomes (Table S4). Discriminant validity was established through a small correlation with concern about effects of COVID-19 on the heart. The VAS of FoRP had a stronger association with the FCRPI total score compared to the FoP-Q-SF, demonstrating that the FCRPI has higher content validity than the FoP-Q-SF in cardiac populations

### Discriminative accuracy of the FCRPI

The FCRPI total score provided excellent discriminative accuracy for determining clinically significant cardiac distress relative to the established CDI-SF cut-off score (≥ 13), with an AUC of 0.91 (*95%CI*: 0.87–0.95; figure S1). A FCRPI total score of 39 was the optimal cut-off to indicate significant distress, providing 89% sensitivity and 79% specificity. In the current sample, *n* = 102 (42%) scored above this cut-off.

## Discussion

The Fear of Cardiac Disease Recurrence and Progression Inventory (FCRPI) is a comprehensive measure of FoRP in cardiac patients. The development of the FCRPI was guided by best practice in instrument design; an item pool was generated from existing literature, theory, and patient and clinical input, and underwent statistical refinement through EFA and Rasch analysis to form the final inventory. Psychometric testing of the FCRPI demonstrated excellent internal consistency and established concurrent and discriminant validity. A total score of 39 on the FCRPI had excellent discriminative accuracy in identifying the level of FoRP related to clinically significant cardiac distress.

Consistent with existing measures of FoRP developed for transdiagnostic use and in other illness populations, the FCRPI captures the multidimensional nature of FoRP. Seven factors were identified from the EFA. Five factors highlighted the type of fears experienced; those relating to deteriorating health, further treatment, disengagement and loss of agency, impacts on intimacy, and impacts on work and finances. The last two factors highlighted responses to FoRP; avoidance and hyperawareness. This factor structure is supported by existing theory-based conceptualisations of FoRP as a construct that spans multiple domains of patient experiences and evokes maladaptive responses^10,11^. Moreover, compared with transdiagnostic measures for FoRP, the FCRPI uniquely reflects cardiac-specific concerns such as fear of physical activity and exertion, misattribution of bodily sensations to cardiac causes, and avoidance of activities perceived to specifically increase cardiac risk^11^.

### Clinical and research utility of the FCRPI

The FCRPI has utility as an instrument to identify clinically significant cardiac-FoRP and to characterise these experiences in both clinical practice and research investigations. Assessing FoRP in clinical practice is critical for identifying patients at the greatest need of support for FoRP. Without active assessment, FoRP-related concerns are likely to go undetected. Patients report reluctance and discomfort in raising these topics spontaneously and non-mental health clinicians similarly report a lack of confidence in managing these concerns^59,60^. The FCRPI provides an avenue to initiate these discussions and equips health professionals with data with which to tailor discussions and interventions to address the specific needs of each patient. Use of the FCRPI cut-off score can identify patients in greatest need of support for FoRP and facilitate appropriate referral to psychological services.

In addition, the FCRPI offers a pathway to further develop research investigating cardiac-FoRP. To our knowledge, the FCRPI is the first cardiac-specific measure of FoRP to be developed. In taking a comprehensive and condition-specific approach to conceptualising FoRP, the FCRPI demonstrated superior validity in cardiac patients than the most frequently cited transdiagnostic measure in this population, the FoP-Q-SF. While previous research has been limited by the absence of a psychometrically validated cut-off score for measures of FoRP in cardiac samples^11^, the identification of a clinically meaningful cut-off score further enhances the research utility of the FCRPI. Use of the cut-off will enable more accurate classification of individuals experiencing clinically-relevant FoRP and facilitate the identification of predictive factors that can be used to inform the design of targeted interventions.

### Study limitations and future directions

While the FCRPI has strength in its robust and comprehensive development and validation process, there are limitations that must be acknowledged. All data, including cardiac diagnoses, were self-reported and not independently confirmed. The item pool was generated largely from English-language research and tested in a predominately Australian and university-educated sample. Cross-cultural validation in demographically diverse populations is warranted to investigate the applicability of the FCRPI in diverse cardiac populations. While the inventory was designed with a cardiac-specific lens, it did not differentiate between different cardiac presentations. Future research is needed to further stratify these samples by specific cardiac presentations, particularly given that cardiac-FoRP may be heightened in those with cardiac presentations that are more chronic in nature (e.g. heart failure and heart rhythm disturbances) or rarer (e.g. spontaneous coronary artery dissection and Takosubo cardiomyopathy) than those with more acute or common presentations^10^. A measure for fear of atrial fibrillation recurrence is currently in development^13^. A psychometric limitation was the lack of validation of Factor 4 (impacts on intimacy) through Rasch analysis due to the inclusion of only two items. Despite its clinical relevance, the two-item sub-scale may limit its stability in future confirmatory factor analysis. In addition, while the 29-item FCRPI offers comprehensive assessment, its length may act as a barrier to its use clinically^61^. Clinicians could target their use of the FCRPI to high-risk groups, specifically younger patients with comorbid health conditions, and those with higher levels of uncertainty and distress regarding their cardiac health^10^. A short-form version of the FCRPI, currently being developed, will have greater utility for cardiac-population screening in time-constrained healthcare settings and facilitate referral to psychological services.

## Conclusions

Our earlier conceptual work identified the need for a cardiac-specific measure to capture the unique and multifaceted experience of FoRP in cardiac patients. The current study has addressed this need through the development and validation of the Fear of Cardiac Recurrence and Progression Inventory (FCRPI). Developed and validated through best-practice methodology, the FCRPI demonstrated strong reliability, validity, and potential clinical utility. Its seven-factor structure reflects the multidimensional and nuanced nature of cardiac-FoRP, capturing both the content of, and responses to, these concerns. The FCRPI is likely to have utility as both a clinical and research tool to identify and characterise clinically significant cardiac-FoRP, as well as to facilitate development of and referral to tailored psychological care.

## Supplementary Information

Below is the link to the electronic supplementary material.

Supplementary Material 1

Supplementary Material 2

## Data Availability

The data that support the findings of this study are available from the corresponding author, S.T.C, upon reasonable request.

## References

[1] Herschbach, P. et al. Fear of progression in chronic diseases: psychometric properties of the fear of progression questionnaire. *J. Psychosom. Res.* **58**, 505–511. 10.1016/j.jpsychores.2005.02.007 (2005). 16125517 10.1016/j.jpsychores.2005.02.007

[2] Herschbach, P. & Dinkel, A. Fear of progression. *Recent. Results Cancer Res.* **197**, 11–29. 10.1007/978-3-642-40187-9_2 (2014). 24305766 10.1007/978-3-642-40187-9_2

[3] American Psychiatric Association. in Diagnostic and statistical manual of mental disorders. (2022).

[4] Mutsaers, B. et al. Identifying the key characteristics of clinical fear of cancer recurrence: an international Delphi study. *Psychooncology* **29**, 430–436. 10.1002/pon.5283 (2020). 31713279 10.1002/pon.5283

[5] Lebel, S. et al. From normal response to clinical problem: definition and clinical features of fear of cancer recurrence. *Supportive Care Cancer: Official J. Multinational Association Supportive Care Cancer*. **24**, 3265–3268. 10.1007/s00520-016-3272-5 (2016). 10.1007/s00520-016-3272-5 27169703

[6] Simard, S. et al. Fear of cancer recurrence in adult cancer survivors: a systematic review of quantitative studies. *J. Cancer Surviv*. **7**, 300–322. 10.1007/s11764-013-0272-z (2013). 23475398 10.1007/s11764-013-0272-z

[7] Sharpe, L., Michalowski, M., Richmond, B., Menzies, R. E. & Shaw, J. Fear of progression in chronic illnesses other than cancer: A systematic review and meta-analysis of a transdiagnostic construct. *Health Psychol. Rev.* 1–40. 10.1080/17437199.2022.2039744 (2022). 10.1080/17437199.2022.2039744 35132937

[8] Almeida, S. N., Elliott, R., Silva, E. R. & Sales, C. M. D. Fear of cancer recurrence: A qualitative systematic review and meta-synthesis of patients’ experiences. *Clin. Psychol. Rev.* **68**, 13–24. 10.1016/j.cpr.2018.12.001 (2019). 30617013 10.1016/j.cpr.2018.12.001

[9] Pradhan, P., Sharpe, L. & Menzies, R. E. Towards a stepped care model for managing fear of cancer recurrence or progression in cancer survivors. *Cancer Manag Res.* **13**, 8953–8965. 10.2147/cmar.S294114 (2021). 34880676 10.2147/CMAR.S294114 PMC8645945

[10] Clarke, S. T., Murphy, B. M., Hester, R. & Jackson, A. C. Fear of recurrence and progression in people with heart disease: risk factors and implications for emotional support. *Behav. Sci.* **15**, 479 (2025). 40282100 10.3390/bs15040479 PMC12024251

[11] Clarke, S. T. et al. Conceptualizing fear of progression in cardiac patients: advancing our Understanding of the psychological impact of cardiac illness. *Heart Mind* **8, **29-39 (2024).

[12] Jackson, A. C. et al. Unraveling the complexity of cardiac distress: A study of prevalence and severity. *Front. Psychiatry*. **13**, 808904. 10.3389/fpsyt.2022.808904 (2022). 35432039 10.3389/fpsyt.2022.808904 PMC9009040

[13] Anthony, S. et al. Fear of recurrence of atrial fibrillation: translating a cancer fear model to the atrial fibrillation patient experience. *Front. Psychiatry*. **13**, 915327. 10.3389/fpsyt.2022.915327 (2022). 35859607 10.3389/fpsyt.2022.915327 PMC9289241

[14] Clarke, S. T., Murphy, B. M., Hester, R. & Jackson, A. C. How does illness uncertainty impact recovery among patients with cardiac conditions? *Br. J. Card Nurs.* **17**, 1–8 (2022).38812658

[15] Xiong, J., Qin, J. & Gong, K. Association between fear of progression and sleep quality in patients with chronic heart failure: A cross-sectional study. *J. Adv. Nurs.*10.1111/jan.15657 (2023). 36978259 10.1111/jan.15657

[16] Xiong, J., Qin, J., Zheng, G., Gao, Y. & Gong, K. The relationship between symptom perception and fear of progression in patients with chronic heart failure: A multiple mediation analysis. *Eur. J. Cardiovasc. Nurs.*10.1093/eurjcn/zvad024 (2023). 36748202 10.1093/eurjcn/zvad024

[17] Zhen, J. et al. Fear of recurrence in elderly patients with coronary heart disease: the current situation and influencing factors according to a questionnaire analysis. *BMC Cardiovasc. Disord*. **22**, 419. 10.1186/s12872-022-02853-w (2022). 36131233 10.1186/s12872-022-02853-w PMC9494841

[18] Fait, K. et al. Cardiac-disease-induced PTSD and fear of illness progression: capturing the unique nature of disease-related PTSD. *Gen. Hosp. Psychiatry*. **53**, 131–138. 10.1016/j.genhosppsych.2018.02.011 (2018). 29779574 10.1016/j.genhosppsych.2018.02.011

[19] Muschalla, B., Glatz, J. & Linden, M. Heart-related anxieties in relation to general anxiety and severity of illness in cardiology patients. *Psychol. Health Med.* **19**, 83–92. 10.1080/13548506.2013.774428 (2014). 23473360 10.1080/13548506.2013.774428

[20] Li, Y., Li, J., Qin, J., Zhou, S. & Gong, K. The sleep patterns and influencing factors of chronic heart failure patients in china: A latent profile analysis. *Nat. Sci. Sleep.* **17**, 571–581. 10.2147/nss.S509059 (2025). 40231044 10.2147/NSS.S509059 PMC11994472

[21] Chen, C. et al. Fear of progression and quality of life in patients with heart failure: a cross-sectional study on the multiple mediation of psychological distress and resilience. *BMC Nurs.* **24**, 60. 10.1186/s12912-025-02688-8 (2025). 39825270 10.1186/s12912-025-02688-8 PMC11742502

[22] Wu, Y. et al. Fear of progression in Chinese patients after cardiac valve replacement: profiles, influencing factors, and mechanisms. *Eur. J. Cardiovasc. Nurs.* **24**, 422–431. 10.1093/eurjcn/zvae178 (2025). 39799975 10.1093/eurjcn/zvae178

[23] Xu, Y., Ma, H. X., Liu, S. S. & Gong, Q. Correlation among anxiety and depression, fear of disease progression, and social support in coronary heart disease. *World J. Psychiatry*. **14**, 1708–1717. 10.5498/wjp.v14.i11.1708 (2024). 39564166 10.5498/wjp.v14.i11.1708 PMC11572683

[24] Liang, Z. et al. A qualitative study exploring the experiences and needs of fear of disease progression in patients after open-heart surgery. *J. Psychosom. Res.* **188**, 111980. 10.1016/j.jpsychores.2024.111980 (2025). 39549654 10.1016/j.jpsychores.2024.111980

[25] Mehnert, A., Herschbach, P., Berg, P., Henrich, G. & Koch, U. [Fear of progression in breast cancer patients–validation of the short form of the fear of progression questionnaire (FoP-Q-SF)]. *Z. Psychosom. Med. Psychother.* **52**, 274–288. 10.13109/zptm.2006.52.3.274 (2006). 17156600 10.13109/zptm.2006.52.3.274

[26] Sharpe, L. et al. The development and validation of the worries about recurrence or progression scale (WARPS). *Br. J. Health Psychol.*10.1111/bjhp.12707 (2023). 38040446 10.1111/bjhp.12707

[27] Boateng, G. O., Neilands, T. B., Frongillo, E. A., Melgar-Quiñonez, H. R. & Young, S. L. Best practices for developing and validating scales for Health, Social, and behavioral research: A primer. *Front. Public. Health*. **6**, 149–149. 10.3389/fpubh.2018.00149 (2018). 29942800 10.3389/fpubh.2018.00149 PMC6004510

[28] Le Grande, M. R. et al. Development of a short form of the cardiac distress inventory. *BMC Cardiovasc. Disord.* **23**, 408. 10.1186/s12872-023-03439-w (2023). 37596516 10.1186/s12872-023-03439-w PMC10439557

[29] Mishel, M. H. The measurement of uncertainty in illness. *Nursing research* (1981). 6912987

[30] Kroenke, K., Spitzer, R. L. & Williams, J. B. The PHQ-9: validity of a brief depression severity measure. *J. Gen. Intern. Med.* **16**, 606–613 (2001). 11556941 10.1046/j.1525-1497.2001.016009606.x PMC1495268

[31] Spitzer, R. L., Kroenke, K., Williams, J. B. & Lowe, B. A brief measure for assessing generalized anxiety disorder: the GAD-7. *Arch. Intern. Med.* **166**, 1092–1097. 10.1001/archinte.166.10.1092 (2006). 16717171 10.1001/archinte.166.10.1092

[32] Weathers, F. W. et al. *The PTSD Checklist for DSM-5*, (2013). www.ptsd.va.gov.

[33] Harris, P. A. et al. Research electronic data capture (REDCap)—a metadata-driven methodology and workflow process for providing translational research informatics support. *J. Biomed. Inform.* **42**, 377–381 (2009). 18929686 10.1016/j.jbi.2008.08.010 PMC2700030

[34] Jackson, A. C. et al. The cardiac distress inventory: A new measure of psychosocial distress associated with an acute cardiac event. *BMC Cardiovasc. Disord.* **22**, 460. 10.1186/s12872-022-02897-y (2022). 36329396 10.1186/s12872-022-02897-y PMC9633013

[35] Tabachnick, B. G. & Fidell, L. S. *Using Multivariate Statistics* (Pearson, 2013).

[36] Costello, A. B. & Osborne, J. Best practices in exploratory factor analysis: four recommendations for getting the most from your analysis. *Practical Assess. Res. Evaluation*. **10**, 1–9 (2005).

[37] Timmerman, M. E. & Lorenzo-Seva, U. Dimensionality assessment of ordered polytomous items with parallel analysis. *Psychol. Methods*. **16**, 209–220. 10.1037/a0023353 (2011). 21500916 10.1037/a0023353

[38] Holgado–Tello, F. P., Chacón–Moscoso, S. & Barbero–García, I. Vila–Abad, E. Polychoric versus pearson correlations in exploratory and confirmatory factor analysis of ordinal variables. *Qual. Quant.* **44**, 153–166. 10.1007/s11135-008-9190-y (2010).

[39] Fabrigar, L. R., Wegener, D. T., MacCallum, R. C. & Strahan, E. J. Evaluating the use of exploratory factor analysis in psychological research. *Psychol. Methods*. **4**, 272–299. 10.1037/1082-989X.4.3.272 (1999).

[40] Tabachnick, B. G., Fidell, L. S. & Ullman, J. B. *Using multivariate statistics*. Vol. 6pearson Boston, MA, (2013).

[41] Finch, W. H. Using fit statistic differences to determine the optimal number of factors to retain in an exploratory factor analysis. *Educ. Psychol. Meas.* **80**, 217–241. 10.1177/0013164419865769 (2020). 32158020 10.1177/0013164419865769 PMC7047263

[42] Linacre, J. M. *Correlations: point-biserial, point-measure, residual*, https://winsteps.com/winman/correlations.htm%3E (n.d).

[43] Boone, W. J. & Staver, J. R. *In Advances in Rasch Analyses in the Human Sciences* (Springer, 2020).

[44] Wright, B. Unidimensionality coefficient. *Rasch Meas. Trans.* **8**, 385 (1994).

[45] Marais, I. in *Rasch Models in Health* 111–130 (2012).

[46] Bond, T. G. & Fox, C. M. *Applying the Rasch Model: Fundamental Measurement in the Human Sciences* (Psychology, 2013).

[47] Linacre, J. M. Optimizing rating scale category effectiveness. *J. Appl. Meas.* **3**, 85–106 (2002). 11997586

[48] Fisher, W. Jr & Reliability Separation, Strata Statistics. *Measurement Transactions* (1992).

[49] Taber, K. S. The use of cronbach’s alpha when developing and reporting research instruments in science education. *Res. Sci. Educ.* **48**, 1273–1296. 10.1007/s11165-016-9602-2 (2018).

[50] Linacre, J. M. *DIF - DPF - DTF - bias - interactions concepts*, < (2023). https://www.winsteps.com/winman/difconcepts.htm

[51] Mokkink, L. B. et al. The COSMIN checklist for evaluating the methodological quality of studies on measurement properties: A clarification of its content. *BMC Med. Res. Methodol.* **10**10.1186/1471-2288-10-22 (2010). 10.1186/1471-2288-10-22 PMC2848183 20298572

[52] Hayes, A. F. & Coutts, J. J. Use Omega rather than cronbach’s alpha for estimating reliability. *But… Communication Methods Measures*. **14**, 1–24. 10.1080/19312458.2020.1718629 (2020).

[53] Çorbacıoğlu, Ş., Aksel, G. & K. & Receiver operating characteristic curve analysis in diagnostic accuracy studies: A guide to interpreting the area under the curve value. *Turk. J. Emerg. Med.* **23**, 195–198. 10.4103/tjem.tjem_182_23 (2023). 38024184 10.4103/tjem.tjem_182_23 PMC10664195

[54] Fluss, R., Faraggi, D. & Reiser, B. Estimation of the Youden index and its associated cutoff point. *Biometrical Journal: J. Math. Methods Biosci.* **47**, 458–472 (2005). 10.1002/bimj.200410135 16161804

[55] Polit, D. F. & Beck, C. T. *Essentials of Nursing Research: Appraising Evidence For Nursing Practice, 9th Edition*Wolters Kluwer,. (2018).

[56] Chen, W. H. et al. Is Rasch model analysis applicable in small sample size pilot studies for assessing item characteristics? An example using PROMIS pain behavior item bank data. *Qual. Life Research: Int. J. Qual. Life Aspects Treat. Care Rehabilitation*. **23**, 485–493. 10.1007/s11136-013-0487-5 (2014). 10.1007/s11136-013-0487-5 23912855

[57] Worthington, R. L. & Whittaker, T. A. Scale development research: A content analysis and recommendations for best practices. *Couns. Psychol.* **34**, 806–838. 10.1177/0011000006288127 (2006).

[58] Hair, J. F., Black, W. C. & Babin, B. J. *Multivariate Data Analysis: A Global Perspective*Pearson Education,. (2010).

[59] Ozakinci, G., Swash, B., Humphris, G., Rogers, S. N. & Hulbert-Williams, N. J. Fear of cancer recurrence in oral and oropharyngeal cancer patients: an investigation of the clinical encounter. *Eur. J. Cancer Care*. **27**10.1111/ecc.12785 (2018). 10.1111/ecc.12785 29024186

[60] Liu, J. J., Butow, P. & Beith, J. Systematic review of interventions by non-mental health specialists for managing fear of cancer recurrence in adult cancer survivors. *Supportive Care Cancer: Official J. Multinational Association Supportive Care Cancer*. **27**, 4055–4067. 10.1007/s00520-019-04979-8 (2019). 10.1007/s00520-019-04979-8 31286237

[61] Jackson, A. C., Grande, L., Higgins, M. R., Rogerson, R. O., Murphy, B. M. & M. & Psychosocial screening and assessment practice within cardiac rehabilitation: A survey of cardiac rehabilitation coordinators in Australia. *Heart Lung Circ.* **26**, 64–72. 10.1016/j.hlc.2016.04.018 (2017). 27283446 10.1016/j.hlc.2016.04.018

