# Supplementary material for: The development and validation of a clinical measurement tool for fear of recurrence and progression in cardiac patients
Source: Sci Rep. 2026 Mar 16;16:13725. doi: 10.1038/s41598-026-40353-5 (PMC13125506; doi:10.1038/s41598-026-40353-5)
Supplement: Supplementary file 1 — Supplementary Material 1 [file 41598_2026_40353_MOESM1_ESM.pdf]

**Supplemental Material: The Development and Validation of a Clinical Measurement Tool for Fear of Recurrence and Progression in Cardiac Patients**

**Page 2-4:** Supplementary description of measures

**Page 5-6:** Table 1: The Fear of Cardiac Recurrence and Progression Inventory (FCRPI) Item Pool with Endorsement Rates

**Page 7:** Table 2. Factor Correlations

**Page 8:** Table 3. Items removed from Rasch analyses

**Page 9:** Table 4. Correlations between FCRPI and validation measures

**Page 10:** Figure 1. Area under the curve graph from receiver operating characteristic analysis

**Supplementary description of measures**

Basic demographic, psychosocial and medical information was collected. This included a question addressing COVID-19 concern for means of assessing discriminant validity, in which participants were asked to rate '*How concerned are you about the effects of COVID-19/long COVID-19 on your heart'* on a scale of 0-3 where 0 = '*not at all*', 1 = '*slightly*', 2 = '*moderately*', 3 = '*extremely*'. Two FoRP measures were included for validation purposes. i) a *Visual Analogue Scale* (VAS) that required participants to rate their fear of their cardiac condition progressing or having recurrent events on a scale of 0 to 10, using either a slider (online) or thermometer (hardcopy). This VAS was adapted from the one-item Fear of Cancer Recurrence screener (FCR-1r)[1], which has demonstrated good validity and reliability in cancer patients[1,2]. ii) the 12-item *Fear of Progression Questionnaire Short Form (FoP-Q-SF)*[3], a transdiagnostic measure of FoRP which has demonstrated good reliability and validity in breast cancer cohorts[3]. Neither FoRP measure has been validated for use in cardiac samples. Cardiac distress was measured through the 12-item *Cardiac Distress Inventory Short Form (CDI-SF)*[4] which has shown excellent internal consistency and good convergent and discriminant validity in cardiac patients[4]. Illness anxiety was assessed using the 23-item *Mishel's Uncertainty in Illness Scale - Community Form (MUIS-C)*[5], designed for use in non-hospitalised chronically ill people, having demonstrated moderate to good internal consistency in cardiac patients with both acute and chronic presentations[6-8]. Depression symptoms were assessed with the 9-item *Patient Health Questionnaire-9 (PHQ-9)*[9] and anxiety symptoms were assessed with the 7-item *Generalised Anxiety Disorder Instrument (GAD-7)*[10], both of which have been validated for use with cardiac patients[11,12]. Post-traumatic stress disorder (PTSD) symptoms were assessed through the 20-item *The PTSD Checklist for DSM-5 (PCL-5)*[13] assessing the 20 PTSD symptoms from the DSM-5.

## References

1 Smith, A. *et al.* Evaluation of the validity and screening performance of a revised single-item fear of cancer recurrence screening measure (FCR-1r). *Psychooncology*, doi:10.1002/pon.6139 (2023).

2 Rudy, L., Maheu, C., Körner, A., Lebel, S. & Gélinas, C. The FCR-1: Initial validation of a single-item measure of fear of cancer recurrence. *Psychooncology* **29**, 788-795, doi:10.1002/pon.5350 (2020).

3 Mehnert, A., Herschbach, P., Berg, P., Henrich, G. & Koch, U. [Fear of progression in breast cancer patients--validation of the short form of the Fear of Progression Questionnaire (FoP-Q-SF)]. *Z Psychosom Med Psychother* **52**, 274-288, doi:10.13109/zptm.2006.52.3.274 (2006).

4 Le Grande, M. R. *et al.* Development of a short form of the Cardiac Distress Inventory. *BMC Cardiovascular Disorders* **23**, 408, doi:10.1186/s12872-023-03439-w (2023).

5 Mishel, M. H. The measurement of uncertainty in illness. *Nursing research* (1981).

6 Carroll, S. L. & Arthur, H. M. A comparative study of uncertainty, optimism and anxiety in patients receiving their first implantable defibrillator for primary or secondary prevention of sudden cardiac death. *International journal of nursing studies* **47**, 836-845, doi:https://doi.org/10.1016/j.ijnurstu.2009.12.005 (2010).

7 Sethares, K. A., Viveiros, J. D. & Ayotte, B. Uncertainty levels differ by physical heart failure symptom cluster. *Applied Nursing Research* **60**, 151435, doi:https://doi.org/10.1016/j.apnr.2021.151435 (2021).

8 McCormick, K. M., Naimark, B. J. & Tate, R. B. Uncertainty, symptom distress, anxiety, and functional status in patients awaiting coronary artery bypass surgery. *Heart & Lung* **35**, 34-45, doi:https://doi.org/10.1016/j.hrtlng.2005.08.002 (2006).

9 Kroenke, K., Spitzer, R. L. & Williams, J. B. The PHQ-9: validity of a brief depression severity measure. *Journal of general internal medicine* **16**, 606-613 (2001).

10 Spitzer, R. L., Kroenke, K., Williams, J. B. & Lowe, B. A brief measure for assessing generalized anxiety disorder: the GAD-7. *Archives of internal medicine* **166**, 1092-1097, doi:10.1001/archinte.166.10.1092 (2006).

11 Stafford, L., Berk, M. & Jackson, H. J. Validity of the Hospital Anxiety and Depression Scale and patient health questionnaire-9 to screen for depression in patients with coronary artery disease. *Gen Hosp Psychiatry* **29**, 417-424 (2007).

12 Conway, A. *et al.* Accuracy of anxiety and depression screening tools in heart transplant recipients. *Applied nursing research : ANR* **32**, 177-181, doi:10.1016/j.apnr.2016.07.015 (2016).

13 Weathers, F. W., Litz, B.T., Keane, T.M., Palmieri, P.A., Marx, B.P., & Schnurr, P.P. . *The PTSD Checklist for DSM-5*, <www.ptsd.va.gov.> (2013).

**Table S1:** The Fear of Cardiac Recurrence and Progression Inventory (FCRPI) Item Pool with Endorsement Rates

| | **Mean (SD)** |
|---|---|
| 1. Your condition getting worse. | 1.39 (0.93) |
| 2. Having another heart event. | 1.54 (0.93) |
| 3. Dying. | 1.27(1.00) |
| 4. Developing other medical problems. | 1.44 (0.96) |
| 5. Your condition impacting your intimate relationships. | 1.03 (1.09) |
| 6. Never getting back to the person you used to be. | 1.58 (1.11) |
| 7. Other people being unable to cope if something happens to you. | 1.36 (1.07) |
| 8. Your general health and functioning declining. | 1.71 (0.96) |
| 9. Not having access to the health care you might need. | 0.98 (1.00) |
| 10. Physical activity leading to another heart event. | 1.14 (0.94) |
| 11. Ageing. | 1.22 (0.95) |
| 12. Receiving permanent scarring from future heart surgeries or removal of veins. | 0.56 (0.86) |
| 13. Losing control over your life. | 1.46 (1.02) |
| 14. Losing capacity for sexual activity. | 0.88 (1.02) |
| 15. Needing to go back to hospital. | 1.22 (1.01) |
| 16. Needing more procedures or surgery. | 1.38 (1.01) |
| 17. Needing to take more medications. | 1.37 (1.05) |
| 18. Being unable to plan for the future. | 1.32 (1.04) |
| 19. Being unable to get help on time. | 1.20 (1.03) |
| 20. Being unable to afford future medical treatments. | 0.96 (1.03) |
| 21. Becoming unable to fulfil your roles at home. | 1.27 (0.99) |
| 22. Becoming unable to fulfil your roles at work. | 0.81 (1.11) |
| 23. Becoming unable to cope effectively. | 1.29 (0.96) |
| 24. Becoming unable to support yourself financially. | 1.14 (1.11) |

| | |
|---|---|
| 25. Becoming unable able to work. | 0.93 (1.11) |
| 26. Becoming unable to engage in activities you enjoy. | 1.68 (1.01) |
| 27. Becoming lonely. | 1.02 (1.06) |
| 28. Becoming less of a person. | 1.04 (1.08) |
| 29. Becoming socially isolated. | 1.04 (1.07) |
| 30. Becoming a burden to your family. | 1.43 (1.10) |
| 31. Becoming more withdrawn from friends. | 1.17 (1.06) |
| 32. Avoid activities that make your heart beat faster. | 1.21 (1.03) |
| 33. Avoid physical exertion. | 1.23 (1.05) |
| 34. Avoid stressful situations. | 1.45 (1.00) |
| 35. Avoid thinking about your heart condition. | 1.21 (1.00) |
| 36. Avoid being alone. | 0.53 (0.88) |
| 37. Avoid going far from home. | 0.92 (1.08) |
| 38. Avoid travelling far from your cardiac care team. | 0.67 (0.95) |
| 39. Avoid medical appointments or check-ups. | 0.17 (0.53) |
| 40. Feel overly aware of your heart in your chest. | 1.42 (1.06) |
| 41. Feel overly aware of sensations in your body. | 1.51 (1.00) |
| 42. Monitor your heart rate. | 1.55 (1.03) |
| 43. Feel worried that you are having another event when you have chest discomfort, or when your heartbeat is fast or irregular. | 1.47 (0.96) |
| 44. Feel worried that your condition is getting worse when you notice changes in your body, such as feeling more fatigued, short of breath, or retaining more fluid. | 1.55 (1.00) |

**Table S2.** Factor Correlations from EFA

| | Factor 1 | Factor 2 | Factor 3 | Factor 4 | Factor 5 | Factor 6 | Factor 7 |
|---|---|---|---|---|---|---|---|
| Factor 1 | 1.00 | 0.43 | 0.50 | 0.40 | 0.39 | 0.40 | 0.20 |
| Factor 2 | 0.43 | 1.00 | 0.33 | 0.50 | 0.50 | 0.33 | 0.12 |
| Factor 3 | 0.50 | 0.33 | 1.00 | 0.33 | 0.41 | 0.28 | 0.19 |
| Factor 4 | 0.40 | 0.50 | 0.33 | 1.00 | 0.38 | 0.35 | 0.17 |
| Factor 5 | 0.39 | 0.50 | 0.41 | 0.38 | 1.00 | 0.31 | 0.23 |
| Factor 6 | 0.40 | 0.33 | 0.28 | 0.35 | 0.31 | 1.00 | 0.18 |
| Factor 7 | 0.20 | 0.12 | 0.20 | 0.17 | 0.23 | 0.18 | 1.00 |

**Factor 1 -** deteriorating health, **Factor 2 -** further treatment, **Factor 3** - disengagement and loss of agency, **Factor 4** - impacts on intimacy, **Factor 5 -** impacts on work and finances, **Factor 6 -** avoidance **Factor 7 -** hyperawareness

**Table S3.** Items removed from Rasch analyses

| Factor | Item | Reason for removal |
|---|---|---|
| 1 | Retain all items | |
| 2 | 12: Receiving permanent scarring from future heart surgeries and veins | Issues with monotonicity |
| | 19: Being unable to get help on time | Significant DIF between different age and education groups |
| 3 | 23: Becoming unable to cope effectively | Placement on Wright Map |
| | 27: Becoming lonely | Issues with dimensionality and local independence |
| | 28: Becoming less of a problem | Placement on Wright Map and issues with dimensionality |
| 5 | 20: Being unable to afford future medical treatment | Issues with dimensionality and significant DIF between age and education levels |
| 6 | Retain all items | |
| 7 | 42: Monitor your heart rate | Issues with monotonicity, placement on the Wright Map, and item fit statistics |

Description of items removed and reasons for removal from Rasch analyses

**Table S4.** Correlations between FCRPI and validation measures

| | | FCRPI total | FoPQ-SF | FoRP VAS |
|---|---|---|---|---|
| **FoPQ-SF** | Pearson's r (95% CI) | 0.86 (0.82 – 0.89) | — | — |
| | *p-value* | <.001 | — | — |
| | *N* | 234 | — | — |
| **FoRP VAS** | Pearson's r (95% CI) | 0.75 (0.68 – 0.80) | 0.67 (0.59 – 0.73) | — |
| | *p-value* | <.001 | <.001 | — |
| | *N* | 241 | 235 | — |
| **PCL** | Pearson's r (95% CI) | 0.69 (0.62 – 0.76) | 0.71 (0.63 – 0.77) | 0.54 (0.44 – 0.62) |
| | *p-value* | <.001 | <.001 | <.001 |
| | *N* | 228 | 228 | 230 |
| **PHQ-9** | Pearson's r (95% CI) | 0.69 (0.62 – 0.75) | 0.71 (0.63 – 0.77) | 0.49 (0.38 – 0.58) |
| | *p-value* | <.001 | <.001 | <.001 |
| | *N* | 226 | 226 | 228 |
| **MUIS-C** | Pearson's r (95% CI) | 0.66 (0.58 – 0.73) | 0.56 (0.46 – 0.64) | 0.53 (0.43 – 0.62) |
| | *p-value* | <.001 | <.001 | <.001 |
| | *N* | 227 | 227 | 229 |
| **GAD-7** | Pearson's r (95% CI) | 0.61 (0.52 – 0.69) | 0.65 (0.57 – 0.72) | 0.50 (0.39 – 0.59) |
| | *p-value* | <.001 | <.001 | <.001 |
| | *N* | 226 | 226 | 228 |
| **COVID-19 Concern** | Pearson's r (95% CI) | 0.39 (0.28 – 0.49) | 0.36 (0.24 – 0.47/l.) | 0.41 (0.30 – 0.51) |
| | *p-value* | <.001 | <.001 | <.001 |
| | *N* | 241 | 235 | 243 |

FoP-Q-SF: fear of progression questionnaire short form; FoRP: fear of recurrence and progression; VAS: visual analogue scale; PCL: The PTSD Checklist for DSM-5; PHQ-9: patient health questionnaire-9; MUIS-C: Mishel's Uncertainty in Illness Scale-Community; GAD-7: General Anxiety Disorder-7; COVID-19: coronavirus disease 2019.

**Figure S1.** Area under the curve graph from receiver operating characteristic analysis

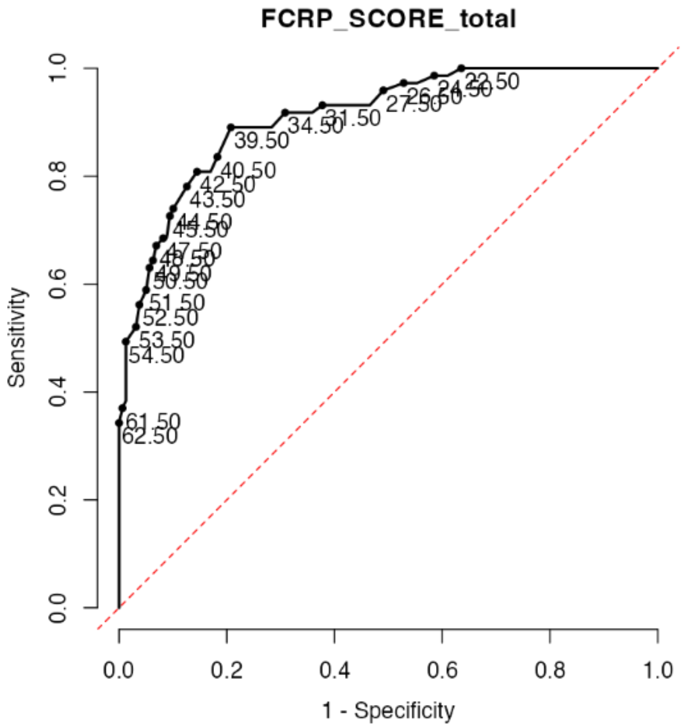


a
