# Supplementary material for: The development and validation of a clinical measurement tool for fear of recurrence and progression in cardiac patients
Source: Sci Rep. 2026 Mar 16;16:13725. doi: 10.1038/s41598-026-40353-5 (PMC13125506; doi:10.1038/s41598-026-40353-5)
Supplement: Supplementary file 2 — Supplementary Material 2 [file 41598_2026_40353_MOESM2_ESM.pdf]

The Fear of Cardiac Recurrence and Progression Inventory (FCRPI)

Living with a heart condition can sometimes be difficult. People often worry about their life and the future. Below is a list of **concerns you may have about your condition becoming worse or having another heart event**.

Please indicate how concerned you are about each of the issues listed below on a scale of 0 to 3. If the statement does not relate to you, please choose "not at all".

**In regard to your heart condition, how concerned are you about:**	**Not at all**	**Slightly**	**Moderately**	**Extremely**
1. Your condition getting worse.	0	1	2	3
2. Having another heart event.	0	1	2	3
3. Dying.	0	1	2	3
4. Developing other medical problems.	0	1	2	3
5. Your condition impacting your intimate relationships.	0	1	2	3
6. Your general health and functioning declining.	0	1	2	3
7. Not having access to the health care you might need.	0	1	2	3
8. Physical activity leading to another heart event.	0	1	2	3
9. Losing control over your life.	0	1	2	3
10. Losing capacity for sexual activity.	0	1	2	3
11. Needing to go back to hospital.	0	1	2	3
12. Needing more procedures or surgery.	0	1	2	3
13. Needing to take more medications.	0	1	2	3
14. Becoming unable to fulfil your roles at home.	0	1	2	3
15. Becoming unable to fulfil your roles at work.	0	1	2	3
16. Becoming unable to support yourself financially.	0	1	2	3

17. Becoming unable able to work.	0	1	2	3
18. Becoming unable to engage in activities you enjoy.	0	1	2	3
19. Becoming socially isolated.	0	1	2	3
20. Becoming a burden to your family.	0	1	2	3
**Because of your concerns about having another heart event or your condition getting worse, <u>how often do you:</u>**	**Not at all**	**A little of the time**	**Some of the time**	**A lot of the time**
21. Avoid activities that make your heart beat faster.	0	1	2	3
22. Avoid stressful situations.	0	1	2	3
23. Avoid being alone.	0	1	2	3
24. Avoid going far from home.	0	1	2	3
25. Avoid medical appointments or check-ups.	0	1	2	3
26. Feel overly aware of your heart in your chest.	0	1	2	3
27. Feel overly aware of sensations in your body.	0	1	2	3
28. Feel worried that you are having another event when you have chest discomfort, or when your heartbeat is fast or irregular.	0	1	2	3
29. Feel worried that your condition is getting worse when you notice changes in your body, such as feeling more fatigued, short of breath, or retaining more fluid.	0	1	2	3

Factors:

1 Deteriorating health (6 items: 1, 2, 3, 4, 8, 10)

2 Further treatment (4 items: 9, 15, 16, 17)

3 Disengagement and loss of agency (5 items: 13, 21, 26, 29, 30)

4 Impacts on intimacy (2 items: 5, 14)

5 Impacts on work and finances (3 items: 22, 24, 25)

6 Avoidance (5 items: 33, 34, 36, 37, 38)

7 Hyperawareness (4 items: 40, 41, 43, 44)

Scoring:

· Obtain total score by adding all item scores together.

· Possible score range = 0-87.

· Cut-off for significant fear of cardiac recurrence and progression is ≥39.
